# Molecular Therapeutic Approaches for Pediatric Acute Myeloid Leukemia

**DOI:** 10.3389/fonc.2014.00055

**Published:** 2014-03-18

**Authors:** Sarah K. Tasian, Jessica A. Pollard, Richard Aplenc

**Affiliations:** ^1^Division of Oncology, Department of Pediatrics, Children’s Hospital of Philadelphia, University of Pennsylvania, Philadelphia, PA, USA; ^2^Division of Hematology/Oncology, Department of Pediatrics, Seattle Children’s Hospital, University of Washington, Seattle, WA, USA

**Keywords:** acute myeloid leukemia, clinical trial, demethylating agents, monoclonal antibodies, pediatric, precision medicine, tyrosine kinase inhibitors, targeted therapy

## Abstract

Approximately two-thirds of children with acute myeloid leukemia (AML) are cured with intensive multi-agent chemotherapy. However, refractory and relapsed AML remains a significant source of childhood cancer mortality, highlighting the need for new therapies. Further therapy intensification with traditional cytotoxic chemotherapy in pediatric AML is not feasible given the risks of both short-term and long-term organ dysfunction. Substantial emphasis has been placed upon the development of molecularly targeted therapeutic approaches for adults and children with high-risk subtypes of AML with the goal of improving remission induction and minimizing relapse. Several promising agents are currently in clinical testing or late preclinical development for AML, including monoclonal antibodies against leukemia cell surface proteins, kinase inhibitors, proteasome inhibitors, epigenetic agents, and chimeric antigen receptor engineered T cell immunotherapies. Many of these therapies have been specifically tested in children with relapsed/refractory AML in Phase 1 and 2 trials with a smaller number of new agents under Phase 3 evaluation for children with *de novo* AML. Although successful identification and implementation of new drugs for children with AML remain a formidable challenge, enthusiasm for novel molecular therapeutic approaches is great given the potential for significant clinical benefit for children who do not have other curative options.

## Introduction

Acute myeloid leukemia (AML) is a group of genetically heterogeneous diseases that account for approximately 20% of pediatric leukemias with approximately 800 newly diagnosed children and adolescents annually in the United States (U.S.) (1, 2). Current intensive cytotoxic chemotherapy regimens achieve long-term cure in only 60–70% of patients, however, and relapsed AML accounts for more than half of childhood leukemia-related deaths (3). Identification of specific genetic subgroups within AML and correlation with relapse-free and overall survival (RFS and OS, respectively) rates have allowed risk stratification of many patients and have decreased use of adjuvant hematopoietic stem cell transplantation (HSCT) for favorable risk group patients (1, 2). Nonetheless, chemotherapy regimens for AML have remained largely unchanged for the past four decades. Relapsed and chemotherapy-refractory AML is thus an area of unmet clinical need and presents opportunities for the development of novel targeted therapeutic approaches. New treatments for children with AML are clearly indicated to decrease relapse and improve cure rates.

Given the higher incidence of AML in adult patients and the limited success of conventional chemotherapy in maintaining long-term remission in this population, various new agents are under active investigation in early-phase clinical trials conducted within predominantly adult cooperative groups. Many of these novel agents aim to target genetic and molecular alterations hypothesized to be involved in leukemogenesis and/or therapy resistance. However, progress in the development and successful implementation of new therapies has been limited by the underlying biologic heterogeneity of AML, the ability of older patients to tolerate intensive therapy, patients’ associated medical co-morbidities, and rapid disease progression at time of relapse.

There is burgeoning evidence that the inherent biology of AML differs between children and adults, as suggested by the discordant incidences of leukemia-associated genetic alterations, patterns of epigenetic changes, and rates of remission induction (4–8). A comprehensive review of the biologic features and genomic landscape of pediatric AML will be published separately in this series. The relatively higher response rates of children to induction chemotherapy may be partially attributable to host factors, such as the generally superior ability of children to tolerate intensive multi-agent therapy, their lower prevalence of medical co-morbidities, and intensive supportive care measures. Nonetheless, primary chemorefractory disease and post-remission relapses remain significant sources of morbidity and mortality for children with AML, and re-induction attempts are frequently unsuccessful (2, 3). In addition, infectious complications and end-organ dysfunction sequelae of intensive chemotherapy occur frequently. Given these issues, investigation of new agents for high-risk childhood AML remains a high priority. Emphasis has been placed upon the development of molecularly targeted agents for childhood AML that will increase rates of successful remission induction, decrease relapse by targeting leukemia-initiating cells, and minimize therapy-associated complications. Our review highlights the current landscape of novel molecular therapeutic approaches for childhood AML (excluding acute promyelocytic leukemia, APL) in clinical or late preclinical testing (Table 1).

**Table 1:** **Molecular therapeutic agents for pediatric acute myeloid leukemia (AML) in current clinical testing or late preclinical development and related trials for adult AML**.

	Target(s)	Phase of testing	Clinical trial number (pediatric)	Clinical trial number (adult)
**MONOCLONAL ANTIBODIES**				
Brentuximab vedotin	CD30	1, 2	Not applicable	NCT01830777, NCT01461538
Gemtuzumab ozogamicin	CD33	1, 2, 3 (with chemotherapy)	NCT00476541, NCT00372593, NCT00070174 (completed)	NCT00091234, NCT00766116, NCT00893399
	CD33	Compassionate use	NCT01869803	NCT01869803
SGN-CD33A	CD33	1	Not applicable	NCT01902329
AMG 330	CD33	Not applicable	Not applicable	Not applicable
Lintuzumab-Ac225 (HuM195)	CD33	1, 2	Not applicable	NCT01756677, NCT00672165
90Y-BC8	CD45	1, 2 (with HSCT)	NCT00119366 (≥16 years)	NCT01300572, NCT00119366
IGN-523	CD98	1	Not applicable	NCT02040506
CSL362	CD123	1	Not applicable	NCT01632852
Ipilimumab	CTLA-4	1	Not applicable	NCT01757639
KB004	EphA3	1, 2	Not applicable	NCT01211691

**TYROSINE KINASE INHIBITORS**				
Lestaurtinib	JAK2, FLT3, TrkA	Not applicable	Not applicable	Not applicable
Midostaurin (PKC412)	CSFR1, FLT3, KIT, PDGFR	1, 2 (with chemotherapy)	NCT00866281	NCT01830361, NCT01883362, NCT01093573, NCT01846624, NCT01477606
Sorafenib	FLT3, Raf kinases, PDGFR, VEGFR	1, 2 (with chemotherapy or post-HSCT)	SJCRH RELHEM (NCT00908167)	NCT01861314, NCT01398501, NCT01534260
		3	COG AAML1031 (NCT01371981)	
Sunitinib	CSF1R, FLT3, KIT, PDGFR, Raf kinases, RET, VEGFR	2	Not applicable	NCT01620216
KW-2449	FLT3	Not applicable	Not applicable	Not applicable
Quizartinib	CSFR1, FLT3, KIT, PDGFR	1, 2, 3	NCT01411267 (completed)	NCT02039726
PLX3397	CSFR1, FLT3, KIT	1, 2	Not applicable	NCT01349049
Linifanib (ABT-869)	FLT3, VEGFR	Not applicable	Not applicable	Not applicable
Crenolanib	FLT3, PDGFR	2	Not applicable	NCT01522469, NCT01657682
ASP2215	FLT3	1, 2	Not applicable	NCT02014558
Sirolimus	mTOR	1, 2 (with chemotherapy)	Not applicable	NCT01822015, NCT01869114, NCT00544999
Temsirolimus	mTOR	2 (with chemotherapy)	Not applicable	NCT01611116
Everolimus	mTOR	1 (with chemotherapy)	Not applicable	NCT01154439
Alisertib (MLN8237)	AURKA	1	Not applicable	NCT01779843
AMG 900	Pan-Aurora kinases	1	Not applicable	NCT01380756
AT9283	Pan-Aurora kinases	1	NCT01431664	Not applicable
**PROTEOSOME INHIBITORS**				
Bortezomib	Proteasome	1, 2, 3 (with chemotherapy)	COG AAML1031 (NCT01371981)	NCT01127009, NCT01371981, NCT01861314, NCT01137747, NCT01204164, NCT01075425, NCT00410423
Carfilzomib	Proteasome	1	Not applicable	NCT01137747, NCT01204164

**EPIGENETIC/DEMETHYLATING AGENTS**				
Decitabine	Methyltransferases	1, 2 (with chemotherapy)	NCT01853228	Numerous
Azacitidine (5-azacytidine)	Methyltransferases	1 (with chemotherapy)	TACL T2011-002 (NCT01861002)	Numerous
Vorinostat	Histone deacetylases	1, 2	NCT01422499	Numerous
Panobinostat	Histone deacetylases	1	TACL T2009-012 (NCT01321346)	Numerous
EPZ-5676	DOT1L methyltransferase	1	Not applicable	NCT01684150
JQ1	Brd4	Not applicable	Not applicable	Not applicable
OXT015	Brd2/3/4	1	Not applicable	NCT01713582

**SELECTIVE INHIBITORS OF NUCLEAR EXPORT**				
KPT-330	CRM1	1	Not applicable	NCT01607892

**CHIMERIC ANTIGEN RECEPTOR T CELL IMMUNOTHERAPY**				
CART33	CD33	1, 2	NCT01864902	NCT01864902
CART123	CD123	Not applicable	Not applicable	Not applicable
Anti-Lewis-Y chimeric antigen receptor	Lewis-Y	1	Not applicable	NCT01716364

*AURKA, aurora kinase A; COG, Children’s Oncology Group; HSCT, hematopoietic stem cell transplantation; SJCRH, St. Jude Children’s Research Hospital; TACL, Therapeutic Advances in Childhood Leukemia; NCT, ClinicalTrials.gov identifier*.

### Antibody therapeutics

Approximately 80% of childhood AML cases express CD33, a glycosylated sialic acid-binding transmembrane receptor protein of the lectin family. High CD33 surface expression is associated with worse outcomes in children and adults with AML (9–12). The potent humanized anti-CD33 monoclonal antibody/calicheamicin conjugate gemtuzumab ozogamicin (GO) has been studied extensively in adults with AML, including APL. Three Phase 2 multi-center trials initially demonstrated second complete remission (CR2) rates of 30% and a favorable safety profile in adults with relapsed AML treated with GO monotherapy (13). These data resulted in accelerated approval of GO in 2000 by the U.S. Food and Drug Administration (FDA) for adults with AML, and other groups have reported similar findings (14, 15).

Gemtuzumab ozogamicin has been specifically studied in pediatric AML via the Children’s Oncology Group (COG) and other consortia. Initial testing of GO monotherapy in the relapsed/refractory setting demonstrated similar pharmacokinetics to those in adult patients and morphologic remission rates of 28–54%, allowing many children to undergo successful subsequent HSCT (16–21). Studies from the St. Jude Children’s Research Hospital (SJCRH) also demonstrated favorable molecular responses in GO-treated children with relapsed/refractory AML treated on the AML02 trial with 13 of 17 patients achieving flow cytometric minimal residual disease levels <0.1% (22). Pilot testing of GO with cytotoxic chemotherapy in children with relapsed/refractory AML also demonstrated the safety and tolerability of combination approaches, and complete remission (CR) was attained in nearly half of these high-risk patients in two independent studies (22, 23).

Based upon these data in the relapse setting, GO and chemotherapy combination regimens were subsequently evaluated in children with *de novo* AML. In the COG pilot trial AAML03P1, CR rates >80% were achieved in children treated with GO and chemotherapy in the induction and post-induction setting (24). In the NOPHO-AML 2004 study, post-consolidation addition of GO to chemotherapy was well-tolerated, but did not alter rates of relapse or OS (25). Most recently, children with *de novo* AML treated with chemotherapy and GO in induction and post-induction on the COG Phase 3 trial AAML0531 experienced decreased rates of relapse and increased event-free survival (EFS) in comparison to children treated with chemotherapy alone (26). Although induction mortality did not differ between treatment arms, a difference in cumulative treatment-related mortality (TRM) approached, but did not reach, statistical significance at rates of 8.6 ± 2.5% for GO/chemotherapy and 5.9 ± 2% for chemotherapy (*p* = 0.09). This increase in TRM was associated with a larger fraction of GO patients with prolonged time to neutrophil recovery in intensification II. Furthermore, this increase in TRM offset gains from the improved relapse rate, and thus no difference in OS was observed between the treatment arms in analyses of all patients. On subgroup analysis, high-risk patients had improved OS and decreased relapse risk (26). Children with CD33-high AML treated with GO and chemotherapy on AAML0531 had disease-free survival rates similar to children with CD33-low AML, suggesting that GO treatment ameliorated the negative outcomes generally associated with high CD33 expression (27). These data highlight the potential for improved treatment efficacy with CD33-targeted agents for certain subgroups of patients, particularly in the setting of high CD33 antigen expression.

Gemtuzumab ozogamicin was withdrawn voluntarily from the U.S. market in 2010 due to concerns for increased induction mortality and lack of efficacy based upon preliminary data from the SWOG-106 trial. Later analyses noted unusually low control arm induction mortality and discordant anthracycline doses between the treatment arms, however, prompting questions regarding the validity of the decision for GO withdrawal (28, 29). More recent mature clinical trial data demonstrate improved outcomes with GO treatment in children and adults with *de novo* AML, particularly in those with favorable cytogenetic characteristics (26, 30–32). As above, GO-treated children treated on AAML0531 did not experience higher induction or overall toxic mortality in comparison to non-GO-treated children (26). A compassionate-use trial for adults and children (≥3 months of age) with relapsed/refractory AML or APL is currently open in the U.S. (NCT01869803) (33). While GO may return to the therapeutic armamentarium in the U.S. for pediatric and adult AML, additional evaluation will likely be required to determine its most appropriate implementation (29, 34).

Alternative anti-CD33 humanized antibody-drug conjugates, such as SGN-CD33A, are under current Phase 1 evaluation in adults with AML given very promising data from initial preclinical testing (NCT01902329) (33). SGN-CD33A is conjugated to a pyrrolobenzodiazepine dimer via a protease-cleavable linker, which has been reported to provide greater drug delivery and stability. Preclinical testing of SGN-CD33A *in vitro* in AML cell lines and *in vivo* in murine xenotransplantation models demonstrated superior leukemia cytotoxicity in comparison to GO. In addition, SGN-CD33A induced greater inhibition of leukemia proliferation and induction of apoptosis in xenograft models of drug-resistant AML (35).

Additional non-drug conjugate antibody approaches in preclinical and clinical testing for cancer include bispecific T cell engaging (BiTE) antibodies, which bind autologous T cells and redirect them specifically against tumor cell antigens. Such approaches have proven successful in early-phase testing for children and adults with leukemia, including the CD19/CD3 BiTE antibody blinatumomab (MT103) for B-precursor ALL (36, 37). Preclinical evaluation of the CD33/CD3 BiTE antibody AMG 330 demonstrated efficient *in vitro* lysis of CD33+ AML cell lines and primary blasts in the presence of human T cells, as well as *in vivo* efficacy in human AML xenograft models. Combination of AMG 330 with epigenetic-targeted therapies may have additional therapeutic efficacy. In preclinical studies, *in vitro* incubation of AML cells with panobinostat or azacitidine increased their CD33 expression, thereby increasing AMG 330-mediated cytotoxicity (38–40). BiTE antibodies for AML are not yet under clinical investigation.

Other antibody-based approaches for AML in early-phase clinical testing include targeting of surface proteins CD30, CD45, CD98, CD123, CTLA-4, or EphA3 (NCT01830777, NCT01756677, NCT02040506, NCT01632852, NCT01757639, NCT01211691) (33). Some of these approaches involve use of radiolabeled antibodies to increase leukemia cytotoxicity (NCT00672165, NCT01300572, NCT01756677) (33, 41). To our knowledge, such strategies have not been specifically evaluated in pediatric AML patients.

### Tyrosine kinase/FLT3 inhibitors

Somatic internal tandem duplication of the gene encoding the fms-like tyrosine kinase receptor-3 (*FLT3*-ITD) with a high mutant:wild-type allelic ratio (>0.4) or point mutations in the FLT3 activation loop of the tyrosine kinase domain (*FLT3*-TKD) occur in 15–20% of pediatric AML patients (42). Similar to adults, children with *FLT3*-ITD AML respond poorly to conventional chemotherapy and have OS of 25–30% even with HSCT (42, 43). Salvage rates of patients with relapsed/refractory *FLT3*-ITD AML are particularly poor with 5-year OS <10% (44). Interestingly, *FLT3*-TKDs do not appear to confer worse outcomes in adults or children with AML (42, 45).

*FLT3*-ITD alterations disrupt the negative regulatory function of the FLT3 protein and facilitate constitutive kinase activation, resulting in perturbed Ras/MAPK, PI3K/Akt/mTOR, and/or JAK/STAT signal transduction and arrest of apoptosis (46, 47). Preclinical studies demonstrate preferential sensitivity of *FLT3*-ITD AML to tyrosine kinase inhibitors (TKIs) that target the FLT3 receptor. In the clinic, TKIs targeting mutant FLT3 have been studied primarily in early-phase trials in adult patients. Modest efficacy of first-generation FLT3 inhibitors (e.g., lestaurtinib, midostaurin, sorafenib, sunitinib) in adults with relapsed AML has been reported, both as monotherapy and in conjunction with cytotoxic chemotherapy (48–52). Many of these first-generation inhibitors are known to target other kinases and wild-type FLT3 protein to some degree and were not designed to inhibit FLT3 preferentially (50, 53–55). Subsequently, TKIs targeting FLT3 more specifically (e.g., KW-2449, PLX3397, quizartinib, linifanib) were developed and are in various stages of preclinical and clinical evaluation as described below (48, 56–59). However, resistance to TKI therapy is well-described and has been attributed to a variety of mechanisms, including development of mutations that interfere structurally with drug binding, alterations in cell survival and signaling pathways, and bone marrow microenvironment factors promoting leukemic survival (60–62). Consequently, third-generation FLT3 TKIs (e.g., crenolanib, ASP2215) have been designed to overcome resistance and have demonstrated preclinical efficacy in drug-resistant *FLT3*-ITD AML with acquired mutations (63–65). These selective FLT3 TKIs are also in early-phase clinical testing for adults with AML (NCT01522469, NCT01657682, NCT02014558) (33). Despite their favorable pharmacokinetic and pharmacodynamic (PD) properties, the majority of next-generation FLT3 TKIs have thus far demonstrated relatively limited anti-AML clinical efficacy as monotherapy and will likely require combination with cytotoxic chemotherapy to achieve maximal therapeutic benefit (55, 66–69).

FLT3 inhibition strategies remain incompletely elucidated and, to date, have undergone relatively limited evaluation in children. Initial preclinical studies demonstrated preferential cytotoxicity of pediatric *FLT3*-ITD AML specimens incubated *in vitro* with lestaurtinib (formerly CEP-701) (70). Lestaurtinib has been better studied clinically in infants with *de novo MLL*-rearranged B-precursor acute lymphoblastic leukemias (ALLs), which overexpress the wild-type FLT3 receptor (71, 72). Lestaurtinib remains under investigation for infant ALL in the current COG Phase 3 trial AALL0631 (NCT00557193) (33, 73). The multi-kinase inhibitor sorafenib was initially evaluated in the COG Phase 1 trial ADVL0413 in children with advanced solid tumors and leukemias; two children with relapsed/refractory *FLT3*-ITD AML achieved ≥CR2 with sorafenib monotherapy on this trial and subsequently underwent HSCT (74). In an institutional case series, three children with relapsed/refractory AML treated with sorafenib and cytotoxic chemotherapy also achieved remission (75). Similarly, 8 of 11 children with relapsed/refractory AML (5 *FLT3*-ITD and 3 *FLT3*-wild-type) treated on a Phase 1 trial at SJCRH with sorafenib, clofarabine, and cytarabine achieved CR or CR with incomplete blood count recovery (CRi) (76).

Recent studies have demonstrated tolerability and improved induction remission in younger adults with *de novo FLT3*-ITD AML treated with sorafenib and chemotherapy, although OS did not differ between the chemotherapy and chemotherapy/sorafenib arms (77). Similarly, a Study Alliance Leukemia trial recently demonstrated no improvement in EFS or OS with sorafenib addition to induction and consolidation chemotherapy for elderly patients with AML (78). However, given the earlier promising clinical data specifically in pediatric AML, the combination of sorafenib and cytotoxic chemotherapy for children with *FLT3*-ITD AML is currently under prospective investigation in the COG Phase 3 trial AAML1031 (NCT01371981) (33). In this trial, children with *FLT3*-ITD AML are non-randomly assigned to treatment with sorafenib in combination with standard chemotherapy for assessment of feasibility (stage 1) and efficacy (stage 2), then taken to HSCT with the best available donor. Interim analyses of AAML1031 data have demonstrated the feasibility and safety of sorafenib with chemotherapy in stage 1, and stage 2 efficacy studies are now underway. A 1-year sorafenib maintenance phase for patients post-HSCT (or post-chemotherapy completion for non-transplanted patients) is underway in AAML1031 based in part upon safety and outcome data from a multi-institutional pilot study (33, 79). Similar sorafenib maintenance studies for adult AML patients are also in progress in the U.S. and in Europe (NCT01398501, NCT01578109, and JA Pollard, personal communication) (33). Although not under current clinical investigation, sorafenib’s c-kit inhibition properties may also have utility for children with *KIT*-mutant AML, which comprises 30% of pediatric core binding factor AML (80).

Studies of the more FLT3-selective second-generation FLT3 TKIs, particularly quizartinib (formerly AC220), have garnered considerable attention for treatment of adults with *FLT3*-ITD AML. Recent Phase 1 and 2 studies of quizartinib in adults with relapsed/refractory AML demonstrated acceptable toxicity profiles and preferential responses of *FLT3*-ITD patients (44, 81). A first-in-children Phase 1 study of quizartinib with chemotherapy for children with relapsed/refractory leukemias was recently conducted via the Therapeutic Advances in Childhood Leukemia (TACL) consortium Phase 1 trial TACL 2009-004 (NCT01411267) (33, 82). The combination regimen was well-tolerated at all doses of quizartinib studied, and near-complete inhibition of phosphorylated FLT3 was observed via PD assays. Three of the six children with *FLT3*-ITD AML achieved CR or CRi, and one of the eight children with *FLT3*-wild-type AML achieved CR (82).

Based upon promising preclinical data in leukemia and clinical testing in solid tumor patients with platelet-derived growth factor receptor (PDGFR) mutations (65, 83), a Phase 2 study of the third-generation FLT3/PDGFR inhibitor crenolanib in adults with AML is also in progress (NCT01522469) (33). Early PD analyses of blood samples from patients enrolled on this trial demonstrate potent FLT3 inhibition with crenolanib treatment (64). Preclinical data also suggest that crenolanib may have efficacy against resistance-conferring *FLT3* point mutations that emerge during TKI therapy, including *FLT3* D835 point mutations. Limited preclinical evaluation of crenolanib incubated with primary leukemia specimens from children with TKI-resistant *FLT3*-ITD/*FLT3* D835 AML demonstrated moderate *in vitro* anti-leukemic activity (63). Most recently, the third-generation FLT3 inhibitor ASP2215 is under Phase 1/2 evaluation in adults with relapsed/refractory AML (NCT02014558) (33). Crenolanib and ASP2215 have not been evaluated clinically in children at this time.

Other TKI approaches for poor-risk adult AML are also under clinical investigation (84). Constitutive activation of the phosphatidylinositide 3-kinase/protein kinase B/mammalian target of rapamycin (PI3K/Akt/mTOR) signal transduction cascade has been well-documented in AML, and PI3K/Akt/mTOR signaling activation often occurs as a downstream sequela of *FLT3*-ITD alterations (85–87). Development of new approaches to inhibit aberrant PI3K pathway signaling is of intense preclinical and clinical interest for AML biologists and clinicians. The mTOR inhibitor rapamycin (also known as sirolimus) and its derivatives are directly cytotoxic to primary AML samples *in vitro* and have proven synergistic with AML-directed cytotoxic chemotherapy *in vivo* in mouse models (88–90). Several early-phase clinical trials in the U.S. and Europe are currently evaluating mTOR inhibition (e.g., sirolimus, temsirolimus, everolimus) in combination with cytotoxic chemotherapy in adults with *de novo* AML (NCT01611116, NCT01154439, NCT01822015, NCT01869114) (33). Although mTOR inhibition in combination with cytotoxic chemotherapy for children with relapsed ALL is under investigation via various consortia (NCT01403415, NCT01523977, NCT01614197) (33), such regimens have not been evaluated in pediatric AML.

Inhibition of the Aurora family of kinases is also under study in patients with relapsed/refractory leukemias. Aurora kinases are critical proteins involved in normal cellular mitosis. Aurora kinase overexpression has been documented in AML and hypothesized to contribute to leukemogenesis (91, 92). Preclinical testing has demonstrated preferential sensitivity of AML cell lines and primary AML specimens to Aurora A and Aurora B kinase inhibitors (91, 92). These agents may have particular therapeutic relevance for patients with acute megakaryoblastic leukemia (AMKL) based upon preclinical data demonstrating increased megakaryocyte polyploidization and AMKL cytotoxicity *in vitro* and *in vivo* with the Aurora kinase A inhibitor alisertib (MLN8237) (93). Clinical trials are in progress to evaluate the safety and/or efficacy of Aurora kinase inhibition as monotherapy or in conjunction with chemotherapy in adults with AML (NCT01779843) (33). Early-phase trial investigation of Aurora kinase inhibitors in children with relapsed/refractory leukemias is also underway in the United Kingdom and the U.S. (NCT01431664, NCT01154816) (33).

### Proteasome inhibitors

The proteasome inhibitor bortezomib has been investigated in the treatment of various malignancies and has demonstrated single-agent efficacy in multiple myeloma. Bortezomib is hypothesized to deplete selectively leukemia-initiating cells and may also augment effects of cytotoxic chemotherapeutic agents (94, 95). Bortezomib has been evaluated specifically in adults with AML in the relapse and *de novo* settings, where it has been reasonably well-tolerated and has demonstrated efficacy in combination with other agents (96–98). Bortezomib and other proteasome inhibitors (e.g., carfilzomib) are under active investigation for adult AML in combination with cytotoxic or demethylating agents in several current clinical trials (NCT01127009, NCT01371981, NCT01861314, NCT01137747, NCT01204164) (33).

Bortezomib was first studied in children with relapsed/refractory solid tumors and leukemia in the COG and TACL consortia (99–102). The subsequent COG Phase 2 trials AALL07P1 and AAML07P1 evaluated bortezomib with intensive re-induction chemotherapy specifically in children with relapsed/refractory ALL and AML, respectively (103, 104). These studies demonstrated safety and tolerability of combination regimens in children with relapsed/refractory leukemias. Among the 38 children with AML treated on AAML07P1 and evaluable for efficacy, 11 CR, 3 CR with incomplete platelet recovery (CRp), and 5 CR with incomplete neutrophil recovery (CRi) were achieved, although OS was not improved with bortezomib addition in comparison to survival data from historical controls (104). Bortezomib in combination with standard AML chemotherapy is currently under investigation for children with *de novo* AML vs. standard AML chemotherapy in the randomized COG Phase 3 trial AAML1031 (NCT01371981) (33).

### Epigenetic/demethylating agents

Genes that regulate DNA methylation and demethylation (e.g., *DNMT3A, IDH1* and *IDH2, TET2*) are commonly mutated in adult AML and myelodysplastic syndromes (MDS), although the frequency of these mutations appears much lower in pediatric AML (105–109). DNA methyltransferase inhibitors (e.g., decitabine, azacitidine/5-azacytidine) are in clinical testing for adults with AML and MDS, and a recent trial demonstrated improved response rates in adults with *DNMT3A*-mutant AML who were treated with decitabine (110). Initial testing of demethylating agents in children with relapsed/refractory AML was performed through the COG, and a recent single-institution study also reported CRs in three of eight children with relapsed/refractory AML treated with decitabine monotherapy (111, 112). Phase 1 studies of azacitidine or decitabine with chemotherapy for children with relapsed/refractory acute leukemias are planned (NCT01861002, NCT01853228) (33).

An additional promising epigenetic strategy for pediatric AML involves inhibition of the DOT1L histone methyltransferase. Preclinical studies of *MLL*-rearranged leukemias have demonstrated a critical role of this enzyme for leukemogenesis, as well as preferential leukemia cytotoxicity with DOT1L inhibition (e.g., EPZ004777) (113–115). A first-in-human Phase 1 study of the DOT1L inhibitor EPZ-5676 for adults with relapsed/refractory MLL-rearranged leukemias recently opened for accrual (NCT01684150) (33).

Another epigenetic strategy involves inhibition of histone deacetylases with agents such as valproic acid, vorinostat, or panobinostat. Vorinostat has proven potentially promising in combination with cytotoxic chemotherapy via early-phase clinical trials for adults with *de novo* AML or MDS (116, 117). A Phase 1/2 study of vorinostat in children with relapsed/refractory cancer, including leukemia, is in progress in Europe (NCT01422499) (33). A Phase 1 study of vorinostat with azacitidine and chemotherapy for children with relapsed ALL is underway via the TACL consortium (NCT01483690) (33), but this combination is not under current investigation in pediatric AML. Early clinical data from adults with AML or MDS treated with vorinostat and demethylating agents demonstrate excellent hematologic responses in many patients (118–121). In one study, clinical responses of patients treated with vorinostat and azacitidine correlated with high pre-treatment methylation levels and demethylation and acetylation during therapy, while patients without evidence of basal hypermethylation or post-treatment demethylation did not respond (119). A Phase 1 trial of panobinostat for children with relapsed/refractory hematologic malignancies is also underway via TACL (NCT01321346) (33). The role of such epigenetic therapies for children with AML remains to be elucidated fully, but early clinical data in adults and children with AML appear encouraging.

The bromodomain and extra-terminal (BET) family of proteins recognize acetylated lysine residues of histone proteins and modulate gene expression via recruitment of transcriptional regulators (122). Inhibition of BET proteins, particularly of Brd4, has demonstrated remarkable anti-tumor activity in a variety of human cancers. Preclinical evaluation of the Brd4 inhibitor JQ1 in AML demonstrated potent *in vitro* and *in vivo* inhibition of leukemia proliferation and elimination of leukemia-initiating cells, likely via suppression of MYC (123, 124). Combination approaches of Brd4 inhibition with TKIs, HDAC inhibitors, or cytotoxic chemotherapy have demonstrated preclinical efficacy in subtypes of AML and remain under active study (125, 126). The preclinical efficacy of the BRD2/3/4 inhibitor OXT015 in AML was also recently reported. *In vitro* incubation of primary AML cells with OXT015 resulted in cell cycle arrest and induction of apoptosis (127). A Phase 1 trial of OTX015 for adults with hematologic malignancies is ongoing in Europe (NCT01713582) (33).

### Selective inhibitors of nuclear export

Localization of cytosolic proteins to the nucleus is critical for normal cellular function. However, the acquired ability of malignant cells to export key nuclear proteins to the cytoplasm is hypothesized to be a major mechanism of treatment resistance. Selective inhibitors of nuclear export, particularly agents targeting the chromosome region maintenance 1 export protein (CRM1, also known as XPO1), have been evaluated in the preclinical setting for various hematologic malignancies, including AML (128, 129). Testing of the CRM1 inhibitors KPT-185 and KPT-251 demonstrated *in vitro* activity against human AML cell lines harboring various genetic alterations and *in vivo* efficacy in AML cell line xenotransplantation models, likely via G1 cell cycle arrest and induction of apoptosis (128, 130). A Phase 1 study of the CRM1 inhibitor KPT-330 is in progress for adults with advanced hematologic malignancies (NCT01607892) (33).

### Chimeric antigen receptor T cell immunotherapy

Rapid progress has been made recently in the development of cancer immunotherapy using human T cells genetically engineered with synthetic chimeric antigen receptors (CARs) to target tumor antigens (131). Current immunotherapeutic approaches for childhood leukemias, including CAR T cell therapy, have been specifically delineated by our colleagues elsewhere in this review series (132). While particular preclinical and early clinical progress has been made with anti-CD19 CAR T cell strategies for B-cell malignancies (133–135), development of anti-AML CAR T cell immunotherapies for the clinic has proven more difficult. Similar to monoclonal antibody therapies for AML (e.g., GO), the paucity of well-characterized, truly leukemia-specific surface antigens in AML has necessitated consideration of CAR T cell AML-targeting strategies that will likely deplete normal hematopoietic cells via “on target/off tumor” effects. Careful consideration of both the leukemia cytotoxicity and the potential for CAR T cell-mediated myeloablation is essential prior to translation of these approaches to the clinic for adults and children with AML.

To date, several candidate AML-associated antigens for CAR T cell approaches have been selected for investigation, including CD33, CD44, CD123, and Lewis-Y (136–141). Preclinical studies of the various anti-AML CAR T cells have generally demonstrated potent *in vitro* cytotoxicity against human AML cell lines and primary specimens (138, 141). Some groups have further observed *in vivo* efficacy of anti-AML CARs using human AML xenograft models (138, 140, 141). However, some reports have noted depletion of normal CD34+ hematopoietic cells by the anti-AML CAR T cells, highlighting the myeloablative potential of such immunotherapeutic approaches for AML (139–141). CAR T cell therapy approaches for AML remain primarily in the preclinical testing phase in the U.S. and Europe, although Phase 1 trials of anti-CD33 CAR T cell therapy (NCT01864902; children ≥5 years of age eligible) and anti-Lewis-Y CAR T cell therapy (NCT01716364; patients ≥18 years of age) for patients with relapsed/refractory AML were recently opened in China and Australia, respectively (33).

## Toward Precision Medicine for Pediatric AML

Despite intensive multi-agent chemotherapy, more than one-third of children with AML will die of chemorefractory or relapsed disease. Further therapy intensification with traditional cytotoxic drugs is unlikely to be tolerated given the current dose intensity of AML chemotherapy. Therefore, new molecular therapeutic approaches that target the AML stem cell responsible for leukemia initiation and progression and that have minimal impact upon normal tissues are needed. Implementation of such targeted strategies will be more likely successful when used in rationally selected combination regimens, which may allow for lower drug dosing (thereby minimizing overlapping toxicities), decrease acquired drug resistance, and increase potential for additive or synergistic cytotoxicity. At a genomics level, significant collaborative efforts are ongoing via the SJCRH–Washington University Pediatric Cancer Genome Project, the National Cancer Institute’s therapeutically applicable research to generate effective treatments (TARGET) AML Project, and other consortia to delineate more precisely various genetic subgroups of pediatric AML and to identify “actionable” lesions for molecularly targeted therapies (8, 142, 143).

One important consideration will be the degree to which results should be extrapolated from adult clinical trial data for children with AML given the inherent, but incompletely understood, differences in biology and in therapeutic responses. Results from early-phase clinical trials of new agents and the number of novel drugs in preclinical development are encouraging, but significant challenges persist in drug development for pediatric oncology. Evaluation of new agents in children with relapsed leukemia in early-phase clinical trials remains particularly challenging given the limited availability of novel drugs for pediatric studies. Moreover, study accrual can be challenging due to rapid disease progression in this population that hinders enrollment. Evaluation of meaningful responses in this setting is also difficult given the use of stringent disease response definitions that limit the number of cycles of therapy administered despite drug mechanisms that may require multiple courses of treatment to achieve optimal response. Given the relative rarity of childhood AML, its biologic heterogeneity, and the prospect of tailored therapeutics for ever-smaller genetic subgroups, collaborative trials across pediatric oncology trial consortia may be a more efficient means by which to identify new molecularly targeted agents for children with specific biologic subtypes of AML. Nonetheless, investigation of promising new therapies is essential to decrease relapse and improve cure for children with AML, and enthusiasm remains high for the development of molecularly targeted approaches in this emerging era of precision medicine.

## Conflict of Interest Statement

The authors declare that the research was conducted in the absence of any commercial or financial relationships that could be construed as a potential conflict of interest.

## References

[1] Meshinchi S Arceci RJ Prognostic factors and risk-based therapy in pediatric acute myeloid leukemia. Oncologist (2007) 12(3):341–55 10.1634/theoncologist.12-3-341 17405900

[2] Pui CH Carroll WL Meshinchi S Arceci RJ Biology, risk stratification, and therapy of pediatric acute leukemias: an update. J Clin Oncol (2011) 29(5):551–65 10.1200/JCO.2010.30.7405 21220611 PMC3071256

[3] Moore AS Kearns PR Knapper S Pearson AD Zwaan CM Novel therapies for children with acute myeloid leukaemia. Leukemia (2013) 27(7):1451–60 10.1038/leu.2013.106 23563239

[4] Creutzig U van den Heuvel-Eibrink MM Gibson B Dworzak MN Adachi S de Bont E Diagnosis and management of acute myeloid leukemia in children and adolescents: recommendations from an international expert panel. Blood (2012) 120(16):3187–205 10.1182/blood-2012-03-362608 22879540

[5] Puumala SE Ross JA Aplenc R Spector LG Epidemiology of childhood acute myeloid leukemia. Pediatr Blood Cancer (2013) 60(5):728–33 10.1002/pbc.24464 23303597 PMC3664189

[6] Radhi M Meshinchi S Gamis A Prognostic factors in pediatric acute myeloid leukemia. Curr Hematol Malig Rep (2010) 5(4):200–6 10.1007/s11899-010-0060-z 20652454

[7] Juhl-Christensen C Ommen HB Aggerholm A Lausen B Kjeldsen E Hasle H Genetic and epigenetic similarities and differences between childhood and adult AML. Pediatr Blood Cancer (2012) 58(4):525–31 10.1002/pbc.23397 22331798

[8] Schuback HL Arceci RJ Meshinchi S Somatic characterization of pediatric acute myeloid leukemia using next-generation sequencing. Semin Hematol (2013) 50(4):325–32 10.1053/j.seminhematol.2013.09.003 24246700

[9] Pollard JA Alonzo TA Loken M Gerbing RB Ho PA Bernstein ID Correlation of CD33 expression level with disease characteristics and response to gemtuzumab ozogamicin containing chemotherapy in childhood AML. Blood (2012) 119(16):3705–11 10.1182/blood-2011-12-398370 22378848 PMC3335378

[10] Hamann PR Hinman LM Hollander I Beyer CF Lindh D Holcomb R Gemtuzumab ozogamicin, a potent and selective anti-CD33 antibody-calicheamicin conjugate for treatment of acute myeloid leukemia. Bioconjug Chem (2002) 13(1):47–58 10.1021/bc010021y 11792178

[11] Walter RB Gooley TA van der Velden VH Loken MR van Dongen JJ Flowers DA CD33 expression and P-glycoprotein-mediated drug efflux inversely correlate and predict clinical outcome in patients with acute myeloid leukemia treated with gemtuzumab ozogamicin monotherapy. Blood (2007) 109(10):4168–70 10.1182/blood-2006-09-047399 17227830 PMC1885511

[12] Jilani I Estey E Huh Y Joe Y Manshouri T Yared M Differences in CD33 intensity between various myeloid neoplasms. Am J Clin Pathol (2002) 118(4):560–6 10.1309/1WMW-CMXX-4WN4-T55U 12375643

[13] Sievers EL Larson RA Stadtmauer EA Estey E Lowenberg B Dombret H Efficacy and safety of gemtuzumab ozogamicin in patients with CD33-positive acute myeloid leukemia in first relapse. J Clin Oncol (2001) 19(13):3244–54 11432892 10.1200/JCO.2001.19.13.3244

[14] Larson RA Sievers EL Stadtmauer EA Lowenberg B Estey EH Dombret H Final report of the efficacy and safety of gemtuzumab ozogamicin (Mylotarg) in patients with CD33-positive acute myeloid leukemia in first recurrence. Cancer (2005) 104(7):1442–52 10.1002/cncr.21326 16116598

[15] Kell WJ Burnett AK Chopra R Yin JA Clark RE Rohatiner A A feasibility study of simultaneous administration of gemtuzumab ozogamicin with intensive chemotherapy in induction and consolidation in younger patients with acute myeloid leukemia. Blood (2003) 102(13):4277–83 10.1182/blood-2003-05-1620 12933575

[16] Reinhardt D Diekamp S Fleischhack G Corbacioglu C Jurgens H Dworzak M Gemtuzumab ozogamicin (Mylotarg) in children with refractory or relapsed acute myeloid leukemia. Onkologie (2004) 27(3):269–72 10.1159/000075606 15249716

[17] Zwaan CM Reinhardt D Zimmerman M Hasle H Stary J Stark B Salvage treatment for children with refractory first or second relapse of acute myeloid leukaemia with gemtuzumab ozogamicin: results of a phase II study. Br J Haematol (2010) 148(5):768–76 10.1111/j.1365-2141.2009.08011.x 19995399

[18] Satwani P Bhatia M Garvin JH Jr George D Dela Cruz F Le Gall J A Phase I study of gemtuzumab ozogamicin (GO) in combination with busulfan and cyclophosphamide (Bu/Cy) and allogeneic stem cell transplantation in children with poor-risk CD33+ AML: a new targeted immunochemotherapy myeloablative conditioning (MAC) regimen. Biol Blood Marrow Transplant (2012) 18(2):324–9 10.1016/j.bbmt.2011.11.007 22079471

[19] Roman E Cooney E Harrison L Militano O Wolownik K Hawks R Preliminary results of the safety of immunotherapy with gemtuzumab ozogamicin following reduced intensity allogeneic stem cell transplant in children with CD33+ acute myeloid leukemia. Clin Cancer Res (2005) 11(19 Pt 2):7164s–70s 10.1158/1078-0432.CCR-1004-0018 16203817

[20] Arceci RJ Sande J Lange B Shannon K Franklin J Hutchinson R Safety and efficacy of gemtuzumab ozogamicin in pediatric patients with advanced CD33+ acute myeloid leukemia. Blood (2005) 106(4):1183–8 10.1182/blood-2004-10-3821 15886328

[21] Buckwalter M Dowell JA Korth-Bradley J Gorovits B Mayer PR Pharmacokinetics of gemtuzumab ozogamicin as a single-agent treatment of pediatric patients with refractory or relapsed acute myeloid leukemia. J Clin Pharmacol (2004) 44(8):873–80 10.1177/0091270004267595 15286091

[22] O’Hear C Inaba H Pounds S Shi L Dahl G Bowman WP Gemtuzumab ozogamicin can reduce minimal residual disease in patients with childhood acute myeloid leukemia. Cancer (2013) 119(22):4036–43 10.1002/cncr.28334 24006085 PMC4271731

[23] Aplenc R Alonzo TA Gerbing RB Lange BJ Hurwitz CA Wells RJ Safety and efficacy of gemtuzumab ozogamicin in combination with chemotherapy for pediatric acute myeloid leukemia: a report from the Children’s Oncology Group. J Clin Oncol (2008) 26(14):2390–3295 10.1200/JCO.2007.13.0096 18467731 PMC4558626

[24] Cooper TM Franklin J Gerbing RB Alonzo TA Hurwitz C Raimondi SC AAML03P1, a pilot study of the safety of gemtuzumab ozogamicin in combination with chemotherapy for newly diagnosed childhood acute myeloid leukemia: a report from the Children’s Oncology Group. Cancer (2012) 118(3):761–9 10.1002/cncr.26190 21766293

[25] Hasle H Abrahamsson J Forestier E Ha SY Heldrup J Jahnukainen K Gemtuzumab ozogamicin as postconsolidation therapy does not prevent relapse in children with AML: results from NOPHO-AML 2004. Blood (2012) 120(5):978–84 10.1182/blood-2012-03-416701 22730539

[26] Gamis A Aplenc R Alonzo TA Sung L Meshinchi S Gerbing RB Gemtuzumab ozogamicin (GO) in children with de novo acute myeloid leukemia (AML) improves event-free survival (EFS) by reducing relapse risk – results from the randomized Phase III Children’s Oncology Group (COG) trial, AAML0531. Blood (2013) 122(21):355

[27] Pollard JA Alonzo TA Gerbing RB Raimondi SC Hirsch B Aplenc R Negative prognostic impact of high CD33 expression is negated with the use of gemtuzumab ozogamicin: a report from the Children’s Oncology Group. Blood (2013) 122(21):49123673863

[28] Petersdorf SH Kopecky KJ Slovak M Willman C Nevill T Brandwein J A phase 3 study of gemtuzumab ozogamicin during induction and postconsolidation therapy in younger patients with acute myeloid leukemia. Blood (2013) 121(24):4854–60 10.1182/blood-2013-01-466706 23591789 PMC3682338

[29] Ravandi F Estey EH Appelbaum FR Lo-Coco F Schiffer CA Larson RA Gemtuzumab ozogamicin: time to resurrect? J Clin Oncol (2012) 30(32):3921–3 10.1200/JCO.2012.43.0132 22987091 PMC4874205

[30] Burnett AK Hills RK Milligan D Kjeldsen L Kell J Russell NH Identification of patients with acute myeloblastic leukemia who benefit from the addition of gemtuzumab ozogamicin: results of the MRC AML15 trial. J Clin Oncol (2011) 29(4):369–77 10.1200/JCO.2010.31.4310 21172891

[31] Burnett AK Russell NH Hills RK Kell J Freeman S Kjeldsen L Addition of gemtuzumab ozogamicin to induction chemotherapy improves survival in older patients with acute myeloid leukemia. J Clin Oncol (2012) 30(32):3924–31 10.1200/JCO.2012.42.2964 22851554

[32] Burnett AK Hills RK Hunter AE Milligan D Kell WJ Wheatley K The addition of gemtuzumab ozogamicin to low-dose Ara-C improves remission rate but does not significantly prolong survival in older patients with acute myeloid leukaemia: results from the LRF AML14 and NCRI AML16 pick-a-winner comparison. Leukemia (2013) 27(1):75–81 10.1038/leu.2012.229 22964882

[33] Health USNIo Clinical Trials. (2013) [cited 2014 Jan 21]. Available from: www.clinicaltrials.gov

[34] Cowan AJ Laszlo GS Estey EH Walter RB Antibody-based therapy of acute myeloid leukemia with gemtuzumab ozogamicin. Front Biosci (2013) 18:1311–34 10.2741/4181 23747885 PMC3683663

[35] Kung Sutherland MS Walter RB Jeffrey SC Burke PJ Yu C Kostner H SGN-CD33A: a novel CD33-targeting antibody-drug conjugate using a pyrrolobenzodiazepine dimer is active in models of drug-resistant AML. Blood (2013) 122(8):1455–63 10.1182/blood-2013-03-491506 23770776

[36] Topp MS Gokbuget N Zugmaier G Degenhard E Goebeler ME Klinger M Long-term follow-up of hematologic relapse-free survival in a phase 2 study of blinatumomab in patients with MRD in B-lineage ALL. Blood (2012) 120(26):5185–7 10.1182/blood-2012-07-441030 23024237

[37] Topp MS Kufer P Gokbuget N Goebeler M Klinger M Neumann S Targeted therapy with the T-cell-engaging antibody blinatumomab of chemotherapy-refractory minimal residual disease in B-lineage acute lymphoblastic leukemia patients results in high response rate and prolonged leukemia-free survival. J Clin Oncol (2011) 29(18):2493–8 10.1200/JCO.2010.32.7270 21576633

[38] Krupka C Kufer P Kischel R Zugmaier G Bogeholz J Kohnke T CD33 target validation and sustained depletion of AML blasts in long-term cultures by the bispecific T-cell-engaging antibody AMG 330. Blood (2014) 123(3):356–65 10.1182/blood-2013-08-523548 24300852

[39] Laszlo GS Gudgeon CJ Harrington KH Dell’Aringa J Newhall KJ Means GD Cellular determinants for preclinical activity of a novel CD33/CD3 bispecific T-cell engager (BiTE) antibody, AMG 330, against human AML. Blood (2014) 123(4):554–61 10.1182/blood-2013-09-527044 24311721 PMC3901068

[40] Aigner M Feulner J Schaffer S Kischel R Kufer P Schneider K T lymphocytes can be effectively recruited for ex vivo and in vivo lysis of AML blasts by a novel CD33/CD3-bispecific BiTE antibody construct. Leukemia (2013) 27(5):1107–15 10.1038/leu.2012.341 23178753

[41] Jurcic JG What happened to anti-CD33 therapy for acute myeloid leukemia? Curr Hematol Malig Rep (2012) 7(1):65–73 10.1007/s11899-011-0103-0 22109628

[42] Meshinchi S Alonzo TA Stirewalt DL Zwaan M Zimmerman M Reinhardt D Clinical implications of FLT3 mutations in pediatric AML. Blood (2006) 108(12):3654–61 10.1182/blood-2006-03-009233 16912228 PMC1895470

[43] Pollard JA Alonzo TA Gerbing RB Woods WG Lange BJ Sweetser DA FLT3 internal tandem duplication in CD34+/CD33− precursors predicts poor outcome in acute myeloid leukemia. Blood (2006) 108(8):2764–9 10.1182/blood-2006-04-012260 16809615 PMC1895585

[44] Takahashi K Kantarjian H Pemmaraju N Andreeff M Borthakur G Faderl S Salvage therapy using FLT3 inhibitors may improve long-term outcome of relapsed or refractory AML in patients with FLT3-ITD. Br J Haematol (2013) 161(5):659–66 10.1111/bjh.12299 23530930 PMC4068703

[45] Bacher U Haferlach C Kern W Haferlach T Schnittger S Prognostic relevance of FLT3-TKD mutations in AML: the combination matters – an analysis of 3082 patients. Blood (2008) 111(5):2527–37 10.1182/blood-2007-05-091215 17965322

[46] Sallmyr A Fan J Datta K Kim KT Grosu D Shapiro P Internal tandem duplication of FLT3 (FLT3/ITD) induces increased ROS production, DNA damage, and misrepair: implications for poor prognosis in AML. Blood (2008) 111(6):3173–82 10.1182/blood-2007-05-092510 18192505

[47] Gu TL Nardone J Wang Y Loriaux M Villen J Beausoleil S Survey of activated FLT3 signaling in leukemia. PLoS One (2011) 6(4):e19169 10.1371/journal.pone.0019169 21552520 PMC3084268

[48] Pratz KW Sato T Murphy KM Stine A Rajkhowa T Levis M FLT3-mutant allelic burden and clinical status are predictive of response to FLT3 inhibitors in AML. Blood (2010) 115(7):1425–32 10.1182/blood-2009-09-242859 20007803 PMC2826764

[49] Wiernik PH FLT3 inhibitors for the treatment of acute myeloid leukemia. Clin Adv Hematol Oncol (2010) 8(6):429–36 20733555

[50] Zhang W Konopleva M Shi YX McQueen T Harris D Ling X Mutant FLT3: a direct target of sorafenib in acute myelogenous leukemia. J Natl Cancer Inst (2008) 100(3):184–98 10.1093/jnci/djm328 18230792

[51] Levis M Ravandi F Wang ES Baer MR Perl A Coutre S Results from a randomized trial of salvage chemotherapy followed by lestaurtinib for patients with FLT3 mutant AML in first relapse. Blood (2011) 117(12):3294–301 10.1182/blood-2010-08-301796 21270442 PMC3069671

[52] Knapper S Burnett AK Littlewood T Kell WJ Agrawal S Chopra R A phase 2 trial of the FLT3 inhibitor lestaurtinib (CEP701) as first-line treatment for older patients with acute myeloid leukemia not considered fit for intensive chemotherapy. Blood (2006) 108(10):3262–70 10.1182/blood-2006-04-015560 16857985

[53] Hu S Niu H Inaba H Orwick S Rose C Panetta JC Activity of the multikinase inhibitor sorafenib in combination with cytarabine in acute myeloid leukemia. J Natl Cancer Inst (2011) 103(11):893–905 10.1093/jnci/djr107 21487100 PMC3110171

[54] Levis M Small D Small molecule FLT3 tyrosine kinase inhibitors. Curr Pharm Des (2004) 10(11):1183–93 10.2174/1381612043452604 15078134

[55] Swords R Freeman C Giles F Targeting the FMS-like tyrosine kinase 3 in acute myeloid leukemia. Leukemia (2012) 26(10):2176–85 10.1038/leu.2012.114 22614177

[56] Zarrinkar PP Gunawardane RN Cramer MD Gardner MF Brigham D Belli B AC220 is a uniquely potent and selective inhibitor of FLT3 for the treatment of acute myeloid leukemia (AML). Blood (2009) 114(14):2984–92 10.1182/blood-2009-05-222034 19654408 PMC2756206

[57] Pratz KW Cortes J Roboz GJ Rao N Arowojolu O Stine A A pharmacodynamic study of the FLT3 inhibitor KW-2449 yields insight into the basis for clinical response. Blood (2009) 113(17):3938–46 10.1182/blood-2008-09-177030 19029442 PMC2673122

[58] Burton E Wong B Zhang J West B Bollag G Habets G The novel inhibitor PLX3397 effectively inhibits FLT3-mutant AML. Blood (2011) 118(21):3632

[59] Wang ES Yee K Koh LP Hogge D Enschede S Carlson DM Phase 1 trial of linifanib (ABT-869) in patients with refractory or relapsed acute myeloid leukemia. Leuk Lymphoma (2012) 53(8):1543–51 10.3109/10428194.2012.660631 22280537

[60] Chu SH Small D Mechanisms of resistance to FLT3 inhibitors. Drug Resist Updat (2009) 12(1–2):8–16 10.1016/j.drup.2008.12.001 19162530 PMC4891941

[61] Weisberg E Sattler M Ray A Griffin JD Drug resistance in mutant FLT3-positive AML. Oncogene (2010) 29(37):5120–34 10.1038/onc.2010.273 20622902

[62] Williams AB Nguyen B Li L Brown P Levis M Leahy D Mutations of FLT3/ITD confer resistance to multiple tyrosine kinase inhibitors. Leukemia (2013) 27(1):48–55 10.1038/leu.2012.191 22858906 PMC3822911

[63] Zimmerman EI Turner DC Buaboonnam J Hu S Orwick S Roberts MS Crenolanib is active against models of drug-resistant FLT3-ITD-positive acute myeloid leukemia. Blood (2013) 122(22):3607–15 10.1182/blood-2013-07-513044 24046014 PMC3837509

[64] Galanis A Ma H Rajkhowa T Ramachandran A Small D Cortes J Crenolanib is a potent inhibitor of FLT3 with activity against resistance-conferring point mutants. Blood (2014) 123(1):94–100 10.1182/blood-2013-10-529313 24227820 PMC3879910

[65] Heinrich MC Griffith D McKinley A Patterson J Presnell A Ramachandran A Crenolanib inhibits the drug-resistant PDGFRA D842V mutation associated with imatinib-resistant gastrointestinal stromal tumors. Clin Cancer Res (2012) 18(16):4375–84 10.1158/1078-0432.CCR-12-0625 22745105

[66] Langdon WY FLT3 signaling and the development of inhibitors that target FLT3 kinase activity. Crit Rev Oncog (2012) 17(2):199–209 10.1615/CritRevOncog.v17.i2.50 22471708

[67] Grunwald MR Levis MJ FLT3 inhibitors for acute myeloid leukemia: a review of their efficacy and mechanisms of resistance. Int J Hematol (2013) 97(6):683–94 10.1007/s12185-013-1334-8 23613268

[68] Altman JK Foran JM Pratz KW Trone D Gammon G Cortes JE Results of a Phase 1 study of quizartinib (AC220, ASP2689) in combination with induction and consolidation chemotherapy in younger patients with newly diagnosed acute myeloid leukemia. Blood (2013) 122(21):623 29139135 10.1002/ajh.24974 PMC6586071

[69] Burnett AK Bowen D Russell N Knapper S Milligan D Hunter AE AC220 (Quizartinib) can be safely combined with conventional chemotherapy in older patients with newly diagnosed acute myeloid leukaemia: experience from the AML18 pilot trial. Blood (2013) 122(21)622

[70] Brown P Meshinchi S Levis M Alonzo TA Gerbing R Lange B Pediatric AML primary samples with FLT3/ITD mutations are preferentially killed by FLT3 inhibition. Blood (2004) 104(6):1841–9 10.1182/blood-2004-03-1034 15166029

[71] Brown P Levis M Shurtleff S Campana D Downing J Small D FLT3 inhibition selectively kills childhood acute lymphoblastic leukemia cells with high levels of FLT3 expression. Blood (2005) 105(2):812–20 10.1182/blood-2004-06-2498 15374878

[72] Brown P Levis M McIntyre E Griesemer M Small D Combinations of the FLT3 inhibitor CEP-701 and chemotherapy synergistically kill infant and childhood MLL-rearranged ALL cells in a sequence-dependent manner. Leukemia (2006) 20(8):1368–76 10.1038/sj.leu.2404277 16761017

[73] Brown P Treatment of infant leukemias: challenge and promise. Hematology Am Soc Hematol Educ Program (2013) 2013:596–600 10.1182/asheducation-2013.1.596 24319237 PMC4729208

[74] Widemann BC Kim A Fox E Baruchel S Adamson PC Ingle AM A phase I trial and pharmacokinetic study of sorafenib in children with refractory solid tumors or leukemias: a Children’s Oncology Group Phase I Consortium report. Clin Cancer Res (2012) 18(21):6011–22 10.1158/1078-0432.CCR-11-3284 22962440 PMC4008314

[75] Watt TC Cooper T Sorafenib as treatment for relapsed or refractory pediatric acute myelogenous leukemia. Pediatr Blood Cancer (2012) 59(4):756–7 10.1002/pbc.23394 22052552

[76] Inaba H Rubnitz JE Coustan-Smith E Li L Furmanski BD Mascara GP Phase I pharmacokinetic and pharmacodynamic study of the multikinase inhibitor sorafenib in combination with clofarabine and cytarabine in pediatric relapsed/refractory leukemia. J Clin Oncol (2011) 29(24):3293–300 10.1200/JCO.2011.34.7427 21768474 PMC3158600

[77] Ravandi F Cortes JE Jones D Faderl S Garcia-Manero G Konopleva MY Phase I/II study of combination therapy with sorafenib, idarubicin, and cytarabine in younger patients with acute myeloid leukemia. J Clin Oncol (2010) 28(11):1856–62 10.1200/JCO.2009.25.4888 20212254 PMC2930809

[78] Serve H Krug U Wagner R Sauerland MC Heinecke A Brunnberg U Sorafenib in combination with intensive chemotherapy in elderly patients with acute myeloid leukemia: results from a randomized, placebo-controlled trial. J Clin Oncol (2013) 31(25):3110–8 10.1200/JCO.2012.46.4990 23897964

[79] Pollard JA Chang BH Cooper TM Gross T Gupta S Ho PA Sorafenib treatment following hematopoietic stem cell transplant in pediatric FLT3/ITD+ AML. Blood (2013) 122(21):3969 10.1002/pbc.25437 25662999

[80] Pollard JA Alonzo TA Gerbing RB Ho PA Zeng R Ravindranath Y Prevalence and prognostic significance of KIT mutations in pediatric patients with core binding factor AML enrolled on serial pediatric cooperative trials for de novo AML. Blood (2010) 115(12):2372–9 10.1182/blood-2009-09-241075 20056794 PMC2845895

[81] Levis MJ Perl AE Dombret H Dohner H Steffen B Rousselot P Final results of a Phase 2 open-label, monotherapy efficacy and safety study of quizartinib (AC220) in patients with FLT3-ITD positive or negative relapsed/refractory acute myeloid leukemia after second-line chemotherapy or hematopoietic stem cell transplantation. Blood (2012) 120(21):673

[82] Cooper TM Malvar J Cassar J Eckroth E Sposto R Gaynon P A Phase I study of AC220 (Quizartinib) in combination with cytarabine and etoposide in relapsed/refractory childhood ALL and AML: a therapeutic advances in childhood leukemia & lymphoma (TACL) study. Blood (2013) 122(21):62423908442

[83] Dai J Kong Y Si L Chi Z Cui C Sheng X Large-scale analysis of PDGFRA mutations in melanomas and evaluation of their sensitivity to tyrosine kinase inhibitors imatinib and crenolanib. Clin Cancer Res (2013) 19(24):6935–42 10.1158/1078-0432.CCR-13-1266 24132921

[84] Smith CC Shah NP The role of kinase inhibitors in the treatment of patients with acute myeloid leukemia. Am Soc Clin Oncol Educ Book (2013) 33:313–8 10.1200/EdBook_AM.2013.33.313 23714533

[85] Park S Chapuis N Tamburini J Bardet V Cornillet-Lefebvre P Willems L Role of the PI3K/AKT and mTOR signaling pathways in acute myeloid leukemia. Haematologica (2010) 95(5):819–28 10.3324/haematol.2009.013797 19951971 PMC2864389

[86] Perl AE Kasner MT Shank D Luger SM Carroll M Single-cell pharmacodynamic monitoring of S6 ribosomal protein phosphorylation in AML blasts during a clinical trial combining the mTOR inhibitor sirolimus and intensive chemotherapy. Clin Cancer Res (2012) 18(6):1716–25 10.1158/1078-0432.CCR-11-2346 22167413 PMC3306511

[87] Martelli AM Evangelisti C Chappell W Abrams SL Basecke J Stivala F Targeting the translational apparatus to improve leukemia therapy: roles of the PI3K/PTEN/Akt/mTOR pathway. Leukemia (2011) 25(7):1064–79 10.1038/leu.2011.46 21436840

[88] Janes MR Fruman DA The in vivo evaluation of active-site TOR inhibitors in models of BCR-ABL+ leukemia. Methods Mol Biol (2012) 821:251–65 10.1007/978-1-61779-430-8_15 22125070

[89] Vu C Fruman DA Target of rapamycin signaling in leukemia and lymphoma. Clin Cancer Res (2010) 16(22):5374–80 10.1158/1078-0432.CCR-10-0480 20826559

[90] Jeong JY Kim KS Moon JS Song JA Choi SH Kim KI Targeted inhibition of phosphatidyl inositol-3-kinase p110beta, but not p110alpha, enhances apoptosis and sensitivity to paclitaxel in chemoresistant ovarian cancers. Apoptosis (2013) 18(4):509–20 10.1007/s10495-013-0807-9 23371322 PMC3604599

[91] Moore AS Blagg J Linardopoulos S Pearson AD Aurora kinase inhibitors: novel small molecules with promising activity in acute myeloid and Philadelphia-positive leukemias. Leukemia (2010) 24(4):671–8 10.1038/leu.2010.15 20147976

[92] Hartsink-Segers SA Zwaan CM Exalto C Luijendijk MW Calvert VS Petricoin EF Aurora kinases in childhood acute leukemia: the promise of aurora B as therapeutic target. Leukemia (2013) 27(3):560–8 10.1038/leu.2012.256 22940834 PMC3593181

[93] Wen Q Goldenson B Silver SJ Schenone M Dancik V Huang Z Identification of regulators of polyploidization presents therapeutic targets for treatment of AMKL. Cell (2012) 150(3):575–89 10.1016/j.cell.2012.06.032 22863010 PMC3613864

[94] Guzman ML Neering SJ Upchurch D Grimes B Howard DS Rizzieri DA Nuclear factor-kappaB is constitutively activated in primitive human acute myelogenous leukemia cells. Blood (2001) 98(8):2301–7 10.1182/blood.V98.8.2301 11588023

[95] Stapnes C Doskeland AP Hatfield K Ersvaer E Ryningen A Lorens JB The proteasome inhibitors bortezomib and PR-171 have antiproliferative and proapoptotic effects on primary human acute myeloid leukaemia cells. Br J Haematol (2007) 136(6):814–28 10.1111/j.1365-2141.2007.06504.x 17341267

[96] Orlowski RZ Voorhees PM Garcia RA Hall MD Kudrik FJ Allred T Phase 1 trial of the proteasome inhibitor bortezomib and pegylated liposomal doxorubicin in patients with advanced hematologic malignancies. Blood (2005) 105(8):3058–65 10.1182/blood-2004-07-2911 15626743

[97] Attar EC Johnson JL Amrein PC Lozanski G Wadleigh M DeAngelo DJ Bortezomib added to daunorubicin and cytarabine during induction therapy and to intermediate-dose cytarabine for consolidation in patients with previously untreated acute myeloid leukemia age 60 to 75 years: CALGB (Alliance) study 10502. J Clin Oncol (2013) 31(7):923–9 10.1200/JCO.2012.45.2177 23129738 PMC3577952

[98] Blum W Schwind S Tarighat SS Geyer S Eisfeld AK Whitman S Clinical and pharmacodynamic activity of bortezomib and decitabine in acute myeloid leukemia. Blood (2012) 119(25):6025–31 10.1182/blood-2012-03-413898 22566605 PMC3383015

[99] Muscal JA Thompson PA Horton TM Ingle AM Ahern CH McGovern RM A phase I trial of vorinostat and bortezomib in children with refractory or recurrent solid tumors: a Children’s Oncology Group phase I consortium study (ADVL0916). Pediatr Blood Cancer (2013) 60(3):390–5 10.1002/pbc.24271 22887890 PMC3511610

[100] Messinger YH Gaynon PS Sposto R van der Giessen J Eckroth E Malvar J Bortezomib with chemotherapy is highly active in advanced B-precursor acute lymphoblastic leukemia: Therapeutic Advances in Childhood Leukemia & Lymphoma (TACL) Study. Blood (2012) 120(2):285–90 10.1182/blood-2012-04-418640 22653976

[101] Messinger Y Gaynon P Raetz E Hutchinson R Dubois S Glade-Bender J Phase I study of bortezomib combined with chemotherapy in children with relapsed childhood acute lymphoblastic leukemia (ALL): a report from the therapeutic advances in childhood leukemia (TACL) consortium. Pediatr Blood Cancer (2010) 55(2):254–9 10.1002/pbc.22456 20582937

[102] Horton TM Pati D Plon SE Thompson PA Bomgaars LR Adamson PC A phase 1 study of the proteasome inhibitor bortezomib in pediatric patients with refractory leukemia: a Children’s Oncology Group study. Clin Cancer Res (2007) 13(5):1516–22 10.1158/1078-0432.CCR-06-2173 17332297

[103] Horton TM Lu X O’Brien MM Borowitz MJ Devidas M Raetz EA Bortezomib reinduction therapy to improve response rates in pediatric ALL in first relapse: a Children’s Oncology Group (COG) study (AALL07P1). J Clin Oncol (2013) 31(Suppl):10003

[104] Horton TM Perentesis J Gamis AS Alonzo TA Gerbing RB Ballard J A Phase 2 study of bortezomib combined with reinduction chemotherapy in children and young adults with recurrent, refractory or secondary acute myeloid leukemia: a Children’s Oncology Group (COG) Study. Blood (2012) 120(21):3580

[105] Damm F Thol F Hollink I Zimmermann M Reinhardt K van den Heuvel-Eibrink MM Prevalence and prognostic value of IDH1 and IDH2 mutations in childhood AML: a study of the AML-BFM and DCOG study groups. Leukemia (2011) 25(11):1704–10 10.1038/leu.2011.142 21647152

[106] Ho PA Alonzo TA Kopecky KJ Miller KL Kuhn J Zeng R Molecular alterations of the IDH1 gene in AML: a Children’s Oncology Group and Southwest Oncology Group study. Leukemia (2010) 24(5):909–13 10.1038/leu.2010.56 20376086 PMC2945692

[107] Ho PA Kopecky KJ Alonzo TA Gerbing RB Miller KL Kuhn J Prognostic implications of the IDH1 synonymous SNP rs11554137 in pediatric and adult AML: a report from the Children’s Oncology Group and SWOG. Blood (2011) 118(17):4561–6 10.1182/blood-2011-04-348888 21873548 PMC3208275

[108] Liang DC Liu HC Yang CP Jaing TH Hung IJ Yeh TC Cooperating gene mutations in childhood acute myeloid leukemia with special reference on mutations of ASXL1, TET2, IDH1, IDH2, and DNMT3A. Blood (2013) 121(15):2988–95 10.1182/blood-2012-06-436782 23365461

[109] Thol F Heuser M Damm F Klusmann JH Reinhardt K Reinhardt D DNMT3A mutations are rare in childhood acute myeloid leukemia. Haematologica (2011) 96(8):1238–40 10.3324/haematol.2011.046839 21685466 PMC3148921

[110] Metzeler KH Walker A Geyer S Garzon R Klisovic RB Bloomfield CD DNMT3A mutations and response to the hypomethylating agent decitabine in acute myeloid leukemia. Leukemia (2012) 26(5):1106–7 10.1038/leu.2011.342 22124213 PMC3696987

[111] Phillips CL Davies SM McMasters R Absalon M O’Brien M Mo J Low dose decitabine in very high risk relapsed or refractory acute myeloid leukaemia in children and young adults. Br J Haematol (2013) 161(3):406–10 10.1111/bjh.12268 23432727

[112] Gore L Macy ME Mechinaud F Narendran A Alvaro F Arndt CAS Interim report of a randomized, open-label, multicenter study to evaluate the safety and efficacy of decitabine as an epigenetic priming agent when combined with induction chemotherapy in pediatric patients (pts) with newly diagnosed acute myelogenous leukemia (AML). Blood (2012) 120(21):1517

[113] Kuhn MWM Hadler M Daigle SR Chen C-W Sinha AU Krivtsov AV Myeloid leukemia cells with MLL partial tandem duplication are sensitive to pharmacological inhibition of the H3K79 methyltransferase DOT1L. Blood (2012) 120(21):1256

[114] Bernt KM Zhu N Sinha AU Vempati S Faber J Krivtsov AV MLL-rearranged leukemia is dependent on aberrant H3K79 methylation by DOT1L. Cancer Cell (2011) 20(1):66–78 10.1016/j.ccr.2011.06.010 21741597 PMC3329803

[115] Daigle SR Olhava EJ Therkelsen CA Majer CR Sneeringer CJ Song J Selective killing of mixed lineage leukemia cells by a potent small-molecule DOT1L inhibitor. Cancer Cell (2011) 20(1):53–65 10.1016/j.ccr.2011.06.009 21741596 PMC4046888

[116] Garcia-Manero G Gore SD Cogle C Ward R Shi T Macbeth KJ Phase I study of oral azacitidine in myelodysplastic syndromes, chronic myelomonocytic leukemia, and acute myeloid leukemia. J Clin Oncol (2011) 29(18):2521–7 10.1200/JCO.2010.34.4226 21576646 PMC3675699

[117] Garcia-Manero G Stoltz ML Ward MR Kantarjian H Sharma S A pilot pharmacokinetic study of oral azacitidine. Leukemia (2008) 22(9):1680–4 10.1038/leu.2008.145 18548103

[118] Stathis A Hotte SJ Chen EX Hirte HW Oza AM Moretto P Phase I study of decitabine in combination with vorinostat in patients with advanced solid tumors and non-Hodgkin’s lymphomas. Clin Cancer Res (2011) 17(6):1582–90 10.1158/1078-0432.CCR-10-1893 21278245

[119] Gore SD Baylin S Sugar E Carraway H Miller CB Carducci M Combined DNA methyltransferase and histone deacetylase inhibition in the treatment of myeloid neoplasms. Cancer Res (2006) 66(12):6361–9 10.1158/0008-5472.CAN-06-0080 16778214

[120] Walter RB Medeiros BC Gardner KM Orlowski KF Gallegos L Scott BL Gemtuzumab ozogamicin in combination with vorinostat and azacitidine in older patients with relapsed or refractory acute myeloid leukemia: a phase I/II study. Haematologica (2014) 99(1):54–9 10.3324/haematol.2013.096545 24142996 PMC4007917

[121] Tan P Wei A Mithraprabhu S Cummings N Liu HB Perugini M Dual epigenetic targeting with panobinostat and azacitidine in acute myeloid leukemia and high-risk myelodysplastic syndrome. Blood Cancer J (2014) 4:e170 10.1038/bcj.2013.68 24413064 PMC3913937

[122] Mertz JA Conery AR Bryant BM Sandy P Balasubramanian S Mele DA Targeting MYC dependence in cancer by inhibiting BET bromodomains. Proc Natl Acad Sci U S A (2011) 108(40):16669–74 10.1073/pnas.1108190108 21949397 PMC3189078

[123] Zuber J Shi J Wang E Rappaport AR Herrmann H Sison EA RNAi screen identifies Brd4 as a therapeutic target in acute myeloid leukaemia. Nature (2011) 478(7370):524–8 10.1038/nature10334 21814200 PMC3328300

[124] Herrmann H Blatt K Shi J Gleixner KV Cerny-Reiterer S Mullauer L Small-molecule inhibition of BRD4 as a new potent approach to eliminate leukemic stem- and progenitor-cells in acute myeloid leukemia AML. Oncotarget (2012) 3(12):1588–99 23249862 10.18632/oncotarget.733 PMC3681497

[125] Fiskus W Sharma S Qi J Valenta JA Schaub LJ Shah B Highly active combination of BRD4 antagonist and histone deacetylase inhibitor against human acute myeloid leukemia (AML) cells. Mol Cancer Ther (2014). 10.1158/1535-7163.MCT-13-0770 24435446

[126] Rodriguez M Fiskus W Sharma S Qi J Valenta JA Schaub LJ Combined therapy with BRD4 antagonist and FLT3 inhibitor exerts synergistic activity against cultured and primary AML blast progenitors expressing FLT-ITD. Blood (2013) 122(21):3821

[127] Braun T Coude MM Berrou J Bertrand S Riveiro E Heralt P Preclinical study of the bromodomain inhibitor OTX015 in acute myeloid (AML) and lymphoid (ALL) leukemias. Blood (2013) 122(21):4218

[128] Etchin J Sanda T Mansour MR Kentsis A Montero J Le BT KPT-330 inhibitor of CRM1 (XPO1)-mediated nuclear export has selective anti-leukaemic activity in preclinical models of T-cell acute lymphoblastic leukaemia and acute myeloid leukaemia. Br J Haematol (2013) 161(1):117–27 10.1111/bjh.12231 23373539 PMC3980736

[129] Lapalombella R Sun Q Williams K Tangeman L Jha S Zhong Y Selective inhibitors of nuclear export show that CRM1/XPO1 is a target in chronic lymphocytic leukemia. Blood (2012) 120(23):4621–34 10.1182/blood-2012-05-429506 23034282 PMC3512237

[130] Etchin J Sun Q Kentsis A Farmer A Zhang ZC Sanda T Antileukemic activity of nuclear export inhibitors that spare normal hematopoietic cells. Leukemia (2013) 27(1):66–74 10.1038/leu.2012.219 22847027 PMC3542631

[131] Barrett DM Singh N Porter DL Grupp SA June CH Chimeric antigen receptor therapy for cancer. Annu Rev Med (2014) 65:333–47 10.1146/annurev-med-060512-150254 24274181 PMC4120077

[132] Shah NN Dave H Wayne AS Immunotherapy for pediatric leukemia. Front Oncol (2013) 3:166 10.3389/fonc.2013.00166 23847759 PMC3696894

[133] Lee DW Barrett DM Mackall C Orentas R Grupp SA The future is now: chimeric antigen receptors as new targeted therapies for childhood cancer. Clin Cancer Res (2012) 18(10):2780–90 10.1158/1078-0432.CCR-11-1920 22589486 PMC4119811

[134] Grupp SA Kalos M Barrett D Aplenc R Porter DL Rheingold SR Chimeric antigen receptor-modified T cells for acute lymphoid leukemia. N Engl J Med (2013) 368(16):1509–18 10.1056/NEJMoa1215134 23527958 PMC4058440

[135] Brentjens RJ Davila ML Riviere I Park J Wang X Cowell LG CD19-targeted T cells rapidly induce molecular remissions in adults with chemotherapy-refractory acute lymphoblastic leukemia. Sci Transl Med (2013) 5(177):177ra38 10.1126/scitranslmed.3005930 23515080 PMC3742551

[136] Peinert S Prince HM Guru PM Kershaw MH Smyth MJ Trapani JA Gene-modified T cells as immunotherapy for multiple myeloma and acute myeloid leukemia expressing the Lewis Y antigen. Gene Ther (2010) 17(5):678–86 10.1038/gt.2010.21 20200563

[137] Tettamanti S Marin V Pizzitola I Magnani CF Giordano Attianese GM Cribioli E Targeting of acute myeloid leukaemia by cytokine-induced killer cells redirected with a novel CD123-specific chimeric antigen receptor. Br J Haematol (2013) 161(3):389–401 10.1111/bjh.12282 23432359

[138] Mardiros A Dos Santos C McDonald T Brown CE Wang X Budde LE T cells expressing CD123-specific chimeric antigen receptors exhibit specific cytolytic effector functions and antitumor effects against human acute myeloid leukemia. Blood (2013) 122(18):3138–48 10.1182/blood-2012-12-474056 24030378 PMC3814731

[139] Marin V Pizzitola I Agostoni V Attianese GM Finney H Lawson A Cytokine-induced killer cells for cell therapy of acute myeloid leukemia: improvement of their immune activity by expression of CD33-specific chimeric receptors. Haematologica (2010) 95(12):2144–52 10.3324/haematol.2010.026310 20713459 PMC2995574

[140] Dutour A Marin V Pizzitola I Valsesia-Wittmann S Lee D Yvon E In vitro and in vivo antitumor effect of anti-CD33 chimeric receptor-expressing EBV-CTL against CD33 acute myeloid leukemia. Adv Hematol (2012) 2012:683065 10.1155/2012/683065 22272203 PMC3261457

[141] Gill S Tasian SK Ruella M Shestova O Li Y Porter DL Efficacy against human acute myeloid leukemia and myeloablation of normal hematopoiesis in a mouse model using chimeric antigen receptor-modified T cells. Blood (2014). 10.1182/blood-2013-09-529537 24596416 PMC3983612

[142] Downing JR Wilson RK Zhang J Mardis ER Pui CH Ding L The pediatric cancer genome project. Nat Genet (2012) 44(6):619–22 10.1038/ng.2287 22641210 PMC3619412

[143] Meshinchi S Ries RE Trevino LR Hampton OA Alonzo TA Farrar JE Identification of novel somatic mutations, regions of recurrent loss of heterozygosity (LOH) and significant clonal evolution from diagnosis to relapse in childhood AML determined by exome capture sequencing – an NCI/COG target AML study. Blood (2012) 120(21):123

